# Hydrogel-Encapsulated Pancreatic Islet Cells as a Promising Strategy for Diabetic Cell Therapy

**DOI:** 10.34133/research.0403

**Published:** 2024-07-04

**Authors:** Zhikun Huan, Jingbo Li, Zhiqiang Luo, Yunru Yu, Ling Li

**Affiliations:** ^1^ Department of Endocrinology, Zhongda Hospital, School of Medicine, Southeast University, Nanjing 210009, China.; ^2^ State Key Laboratory of Bioelectronics, School of Biological Science and Medical Engineering, Southeast University, Nanjing 210096, China.; ^3^ Pharmaceutical Sciences Laboratory, Åbo Akademi University, Turku 20520, Finland.

## Abstract

Islet transplantation has now become a promising treatment for insulin-deficient diabetes mellitus. Compared to traditional diabetes treatments, cell therapy can restore endogenous insulin supplementation, but its large-scale clinical application is impeded by donor shortages, immune rejection, and unsuitable transplantation sites. To overcome these challenges, an increasing number of studies have attempted to transplant hydrogel-encapsulated islet cells to treat diabetes. This review mainly focuses on the strategy of hydrogel-encapsulated pancreatic islet cells for diabetic cell therapy, including different cell sources encapsulated in hydrogels, encapsulation methods, hydrogel types, and a series of accessorial manners to improve transplantation outcomes. In addition, the formation and application challenges as well as prospects are also presented.

## Introduction

Diabetes mellitus (DM) refers to a group of metabolic disorders that are characterized by hyperglycemia [1–3]. Insufficient insulin secretion would result in type 1 diabetes, later-stage type 2 diabetes, and some other specific types of diabetes, where exogenous insulin supplementation is usually required for treatment [4,5]. However, frequent insulin injections not only bring discomfort to patients and reduce their compliance but also fail to reduce the risk of various complications associated with DM [6–9]. Therefore, exploring new options for diabetes treatment and addressing the issues caused by traditional treatment programs has become a new research focus [10–13]. Cell therapy, by contrast, can achieve diabetic glycemic control by restoring endogenous insulin supplementation, and promises to be a new option for treating DM [14–16].

In cell therapy, primary islets, stem cell-derived islets (SC-islets), and pancreatic β cell lines that can produce insulin have all been used for transplantation explorations [9,17–24]. To improve the protection and the longevity of the transplanted cells in the body, strategies to encapsulate the cells with nano-, micro-, and macroscale hydrogel have been continuously discussed [25–28]. To achieve better glycemic control, researchers are exploring the transplantation of devices encapsulating islet cells into various sites, including subcutaneous, abdominal, and subperitoneal, while ensuring the necessary nutrients, oxygen supply, and immune isolation for graft survival in vivo [29–31].

**Fig. 1.**
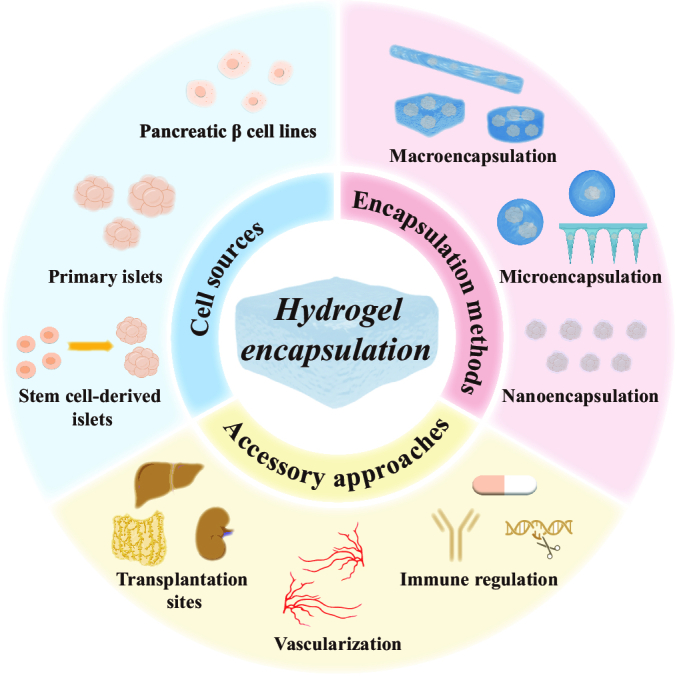
Schematic illustration of different approaches to preparing hydrogel-encapsulated islet cells and their applications in diabetic cell therapy. The hydrogel encapsulation strategy is described in 3 main aspects, including cell sources (pancreatic β cell lines, primary islets, and SC-islets), encapsulation methods (macro-, micro-, and nanoencapsulation), and accessory approaches (optimization of transplantation sites, improvement of vascularization, and immune regulation).

In this review, we present various strategies for hydrogel-encapsulated pancreatic islet cells and their applications in the diabetes therapeutic field, as schemed in Fig. 1. Specifically, the review will be divided into 3 parts. In the first part, we list different sources of insulin-secreting cells being used for transplantation. In the second and third parts, we describe different hydrogel encapsulation methods and some accessory approaches to improve transplantation effects, respectively. Finally, we conclude the applications of this strategy and make a prospective view of these hydrogel-encapsulated islet cells for the treatment of DM.

## Source of Transplanted Islet Cells

The Edmonton protocol, introduced in 2000, represented an important breakthrough in islet transplantation. It moved from isolation techniques in the laboratory to clinical outcomes [32]. With the development of islet isolation techniques and anti-immune rejection therapy protocols, transplantation efficacy has been further improved [33,34]. In 2023, the Food and Drug Administration (FDA) approved the first allogeneic islet cell therapy generated from deceased donor-derived pancreatic cells [35,36]. However, donor shortages remain a limitation to the widespread clinical use of islet transplantation [37,38]. Thus, the exploration of insulin-secreting β cells that can be used for transplantation has become an alternative (Table 1).

**Table 1.** Comparison of different cell sources in islet transplantation

Cell sources	Cell names	Advantages	Disadvantages
Cell lines	MIN-6	Adequate cell numbers Able to aggregate in vitro to form pseudo-islets	Risk of unlimited proliferation in vivo
	β-TC-6		
	NIT-1		
	INS-1		
Primary islets	Islets of Langerhans	Original cellular composition and structure Highly capable of hormone secretion	Donor shortages
SC-islets	ESC	Well-sourced Good plasticity Hormone-secreting ability after induced differentiation	Undesirable heterogeneity Poorer function than primary islets Non-negligible potential tumorigenicity
	MSC		
	iPSC		

With effective tools for studying pancreatic β cell biology, various types of β cell lines are used to investigate pancreatic islet function [39,40]. These cell lines have also been used for transplantation in diabetic animals, such as MIN-6 [41], β-TC-6 [42], NIT-1 [43], INS-1 [44], and others. The advantages of using cell lines are the abundant cell sources, the desired cell function, as well as the possibility to manipulate cells with gene editing tools [45]. By overexpressing the glucagon-like peptide-1 (GLP-1) receptor in INS-1 cells, Chepurny and Holz [46] prompted glucose-dependent insulin biosynthesis and secretion in pancreatic β cells for transplantation. However, the transplantation of cell lines casts the risk for tumorigenesis and hypoglycemia due to unlimited in vivo proliferation [47]. Thus, pancreatic β cell lines are suitable for primary evaluation of transplanting strategies, rather than practical clinical applications.

Islets remain the main source of grafts currently used for diabetes treatment [47]. The isolation and purification of donor pancreatic islets conform to standardized processes in fundamental and clinical research, followed by the transplantation into specific sites in the experimental animals or human recipients [48,49]. However, the quantity and quality of primary pancreatic islets can hardly meet the huge therapeutic needs of numerous diabetic patients [50]. As a result, alternative sources of pancreatic islets have received considerable attention [51]. In addition, the close interactions between pancreatic β cells and other pancreatic cells, such as pancreatic α cells and some pancreatic exocrine cells, suggest the effects of them on establishing functional islets. It is found that pancreatic α cells can be transdifferentiated into insulin-secreting cells but the biological mechanism is unclear, limiting their further applications for diabetes treatment [52–54].

With the discovery of embryonic stem cells (ESCs), mesenchymal stem cells (MSCs), and induced pluripotent stem cells (iPSCs), infinite islets can be produced owing to the virtually unlimited potential of these stem cells for division and differentiation [55–57]. Many groups have successfully induced SC-islets with multicellular components by administering various types of growth factors and transcription factors to stem cells, which have been applied to islet transplantation, organoid chips, and other fields [58–60]. Ideally, SC-islets can be used as a new transplantation source to address the limitation of donor sources due to their similar functions as natural islets [55]. However, the current issues such as the wide variation in maturity and low reproducibility of SC-islets from different batches of stem cells can result in some uncertainty of their treatment effects [38]. Continuous optimization of induction methods and systems is necessary for the production of more structurally and functionally mature SC-islets.

## Strategies for Encapsulating Islet Cells

In clinical islet transplantation attempts, there exist certain challenges such as the necessity for long-term immunosuppressive therapy and posttransplantation cell loss, which limits their widespread adoption in clinical conditions [61]. Islet cell encapsulation technology holds promises in mitigating islet cell loss and minimizing or eliminating immune rejection, making it a crucial approach to improving the survival rate of transplanted islet cells [62]. Ideal materials for islet cell encapsulation should meet the following demands: causing no immune response or excessive fibrosis in recipients, low degradation rate and long in vivo presence, and high biocompatibility as well as long-lasting cell survival and functioning [63]. However, current materials failed to fully meet those criteria. Currently, both natural (e.g., hyaluronic acid, collagen, and sodium alginate) and synthetic [e.g., polyacrylamide and polyethylene glycol (PEG)] hydrogels have been used to encapsulate pancreatic islets [64]. Because the survival and function of the implanted pancreatic islet cells are affected by the diffusion distance between cells and the host after transplantation, the size of the encapsulation devices is taken into consideration during hydrogel design and fabrication. On this basis, the prepared encapsulation devices can be classified into macro-, micro-, and nanoencapsulation [65].

### Macroencapsulation

**Fig. 2.**
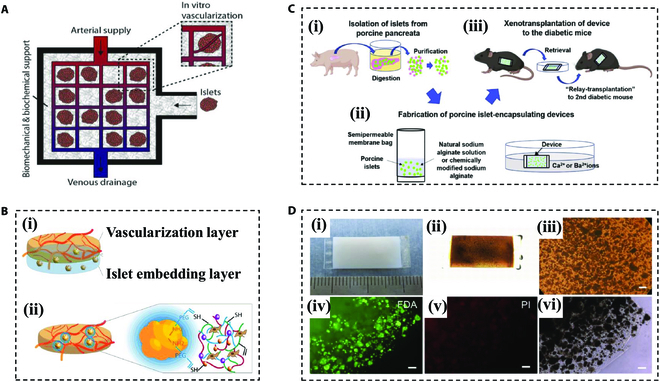
Representative macroencapsulation devices. (A) Diagram of the VIO device. Reproduced with permission [70]. Copyright 2019, Elsevier Ltd. (B) Two strategies for encapsulating pancreatic islets with layered PEGDA hydrogels. (i) PEGDA hydrogel was used for the entire layer of the islet encapsulation. (ii) PEGDA hydrogel coatings were used to apply to the surface of individual pancreatic islets. Reproduced with permission [66]. Copyright 2017, Springer. (C) Schematic representation of the porcine islet wrapping device and in vivo transplantation. Reproduced with permission [71]. Copyright 2022, The Authors. (D) Characterization of alginate semipermeable membrane hydrogels encapsulating pancreatic islets. Scale bars, 200 μm. Reproduced with permission [71]. Copyright 2022, The Authors.

Macroencapsulation refers to the systems that encapsulate a large number of pancreatic islets or islet cells in a moldable hydrogel [66]. The macroencapsulation poses advantages mainly in the huge capacity, which usually requires only one or a few devices to reach the islet requirement for treatment [67,68]. In addition, the large size of these hydrogels makes them easy to remove from the body in case any problem occurs in the hydrogel or the cells [69]. Citro et al. [70] developed a bioengineered vascularized islet organ (VIO) that incorporated a vascular network (Fig. 2A). The treatment quickly reversed high blood glucose levels in diabetic mice and maintained long-standing normoglycemia. In a separate study, Marchioli et al. [66] explored 2-layered structures based on PEG diacrylate (PEGDA) hydrogel encapsulation devices combined with controlled release of vascular endothelial growth factor (VEGF) and basic fibroblast growth factor (bFGF) to achieve morphology as well as the long-term survival of encapsulated pancreatic islets (Fig. 2B). To confirm the retrievability of the macroencapsulation devices, Ajima et al. [71] encapsulated porcine-derived pancreatic islets in a high-stability and immuno-isolated alginate hydrogel, and the device was not only able to improve blood glucose but could even tolerate 2 different xenograft reactions (Fig. 2C and D). In a recent study, Wang et al. [72] used polyether sulfone (PES) as a mechanically supported outer layer, with sodium alginate embedded in the middle with islet mixtures, to achieve blood glucose correction over 90 days.

**Fig. 3.**
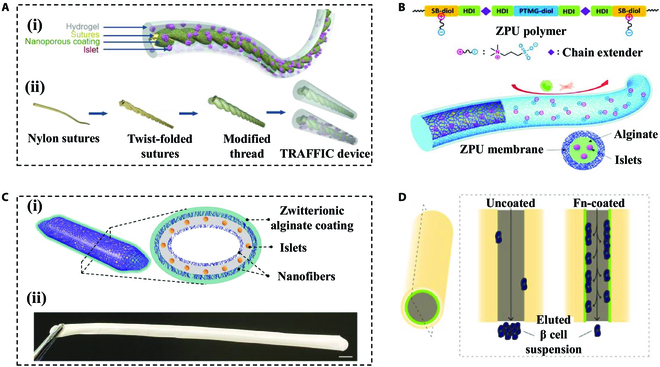
Hydrogel fiber devices for encapsulating islets. (A) Schematic illustration of designing and fabricating the TRAFFIC device. Reproduced with permission [78]. Copyright 2018, published under the PNAS license. (B) Schematic illustration of the chemical structure of the ZPU polymer and islet containing ZPU device. Reproduced with permission [79]. Copyright 2021, Wiley-VCH GmbH. (C) (i) Schematic of the cross-sectional structure of SHIELD encapsulated pancreatic islets. (ii) Representative image of SHIELD. Scale bar, 5 mm. Reproduced with permission [80]. Copyright 2021, Wiley-VCH GmbH. (D) Schematic illustration of hollow porous PES fibers inducing islet cells to adhere to the tubular shell and inhibiting fibrosis. Reproduced with permission [86]. Copyright 2020, American Chemical Society.

Apart from bulk hydrogels, fibers are extensively utilized in islet cell encapsulation and transplantation due to their small diameters, enabling sufficient exchanges of substances and retrievability [73,74]. Sodium alginate, the most commonly chosen graft material, is designed to encapsulate pancreatic islet cells or islet organoids to achieve the glycemic control [75]. The function and activity of encapsulated pancreatic islets can be further enhanced by incorporating hyaluronate, collagen, extracellular matrix (ECM), etc. [73,76,77]. Addressing the mechanical limitations of sodium alginate hydrogels, Ma’s group [78] designed a thread-reinforced alginate fiber for islet encapsulation (TRAFFIC) by crosslinking alginate hydrogels in situ around nanoporous, wettable, calcium-releasing polymer threads (Fig. 3A). The device provided enhanced mechanical properties as well as strong diabetes therapeutic potential when encapsulated around pancreatic islets. Moreover, they also proposed a similar zwitterionic polyurethane (ZPU) device (Fig. 3B) [79] and a safe, hypo-immunoreactive, islet encapsulation, long-term-functional device (SHIELD) (Fig. 3C) [80], which aimed to mitigate foreign body reactions in islet transplantation. These innovations inspired the development of fiber-like hydrogel encapsulation for pancreatic islet cells. Besides, the double crosslinked hydrogels based on sodium alginate by employing hydrogels, such as PEGDA [81], cellulose nanocrystals [82], and hydroxyethyl cellulose [83], have also been generated to improve the mechanical toughness of the hydrogel fibers while maintaining good biocompatibility.

The physical properties of the hydrogel fibers, in addition to their chemical composition, are also important in the regulation of the functions. By optimizing the morphology of the hydrogel fibers, a hollow structure was made to enhance the efficiency of nutrient exchanges, and simulate subsequent vascularized structures, while maintaining the immune isolation of the islet cells [84]. In 1991, studies were exploring the possibility of encapsulating pancreatic islets in hollow fibers with an acrylate copolymer shell, which achieved satisfying results after transplantation in diabetic mice [85]. Since conventional polymers applied in the present devices were poor substrates for cell attachment and could trigger fibrosis, hollow fibers were prepared through semi-porous polyethersulfone (PES) encapsulated membranes and coated with fibronectin on their inner surface to enhance islet cell adhesion (Fig. 3D) [86]. To achieve more islets wrapped within a certain length of fiber, Skrzypek et al. [87] then designed a fiber with multiple holes to promote islet cell capacity for transplantation therapy without prolonging the length and volume of the hydrogel.

**Fig. 4.**
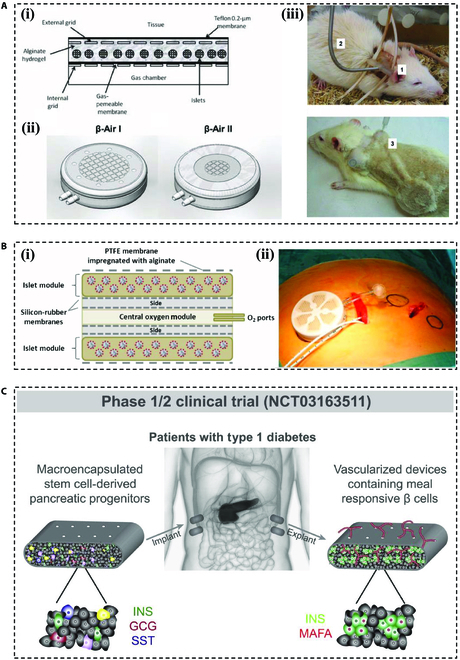
Macroencapsulation devices for clinical trials. (A) Schematic drawings of the 2 “β-AIR” devices with different islet volumes and ventilation, as well as photographs of each of them implanted in mice. Reproduced with permission [88]. Copyright 2013, Cognizant Comm. Corp. (B) (i) Schematic illustration of the chamber system for macroencapsulation of islets. (ii) Picture of the graft as it is prepared for implantation in the human body. Reproduced with permission [89]. Copyright 2013, published under the PNAS license. (C) Schematic diagram of a clinical trial in which the patient with type 1 diabetes was transplanted in vivo with a device containing PEC-01 cells. Reproduced with permission [92]. Copyright 2021, Elsevier Inc.

In addition to the explorations that are still being conducted in animal experiments, a few macroencapsulation devices have been subjected to clinical trials. In 2013, researchers from Israel reported an alginate hydrogel to encase the islets, named the “β-AIR” device. This device consisted of gas chambers, a gas-permeable membrane, an external membrane, and a mechanical support (Fig. 4A) [88]. The device was first validated for glycemic control in diabetic rats [88]. Furthermore, after optimizing the device, they achieved glycemic control by implanting a chamber macroencapsulation system in a patient with type 1 diabetes (Fig. 4B) [89]. Based on the results of these preclinical experiments, they implanted 1-2 “β-AIR” devices in 4 subjects (Clinicaltrials.gov: NCT02064309), which showed that implantation of these devices was safe and the islet cells were viable. Nevertheless, there was only a small amount of circulating C-peptide in the subjects, and metabolic control was not improved in the end [90].

The US company Viacyte, on the other hand, used an immuno-isolated macroencapsulation device (PEC-Encap) to encapsulate stem cell-derived pancreatic precursor cells (PEC-01 cells) for clinical trials (Clinicaltrials.gov: NCT02239354). In this clinical trial, the device demonstrated its good tolerance and capability of generating insulin-secreting cells [91]. In 2017, they optimized the membrane of the device (PEC-Direct) to allow vascularization to penetrate inside the cells. The results suggested the feasibility of successfully realizing a functional vascularized islet encapsulation device in vivo (Fig. 4C; Clinicaltrial.gov: NCT03163511) [92]. In 2023, Vertex Pharmaceuticals initiated phase 1/2 clinical trials in Canada for VX-264, an advanced surgical device designed for the transplantation of human SC-islets encapsulated within a channel array (Clinicaltrials.gov: NCT05791201). This innovative technology aims to address type 1 diabetes with utmost precision and efficacy.

### Microencapsulation

**Fig. 5.**
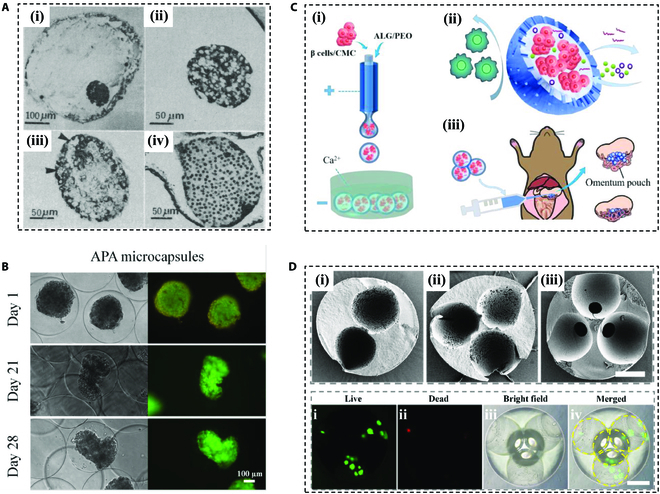
Representative microencapsulation devices. (A) First microencapsulation of pancreatic islets by sodium alginate. Reproduced with permission [95]. Copyright 1980, AAAS. (B) Image of APA microencapsulated INS-1E pseudo-islets. Reproduced with permission [102]. Copyright 2019, Elsevier B.V. (C) Schematic demonstrating the fabrication of the porous microcapsules encapsulating β cells for diabetes treatment. Reproduced with permission [104]. Copyright 2022, The Authors. (D) Scanning electron microscopy (SEM) of the polymeric hydrogel particles and the live/dead staining of the microcarrier. Scale bars, 200 μm. Reproduced with permission [107]. Copyright 2022, Elsevier B.V.

Microencapsulation is the encapsulation of pancreatic islet cells into small-diameter hydrogel-like polymeric microdevices [93,94]. Typically, the hydrogel is employed in microencapsulation to encapsulate single or several islets for transplantation. In 1980, Lim and Sun [95] successfully achieved a 3-week glycemic control in diabetic rats by employing alginate hydrogel microcapsules to encapsulate pancreatic islets for the first time (Fig. 5A). Following this, researchers have endeavored to expand the scope of microencapsulation methodologies [96–98]. For instance, experimentation with agarose gels for microencapsulation has been conducted [99], and xenotransplantation has been explored. After transplantation, the microcapsule-encapsulated islets achieved normoglycemia for a maximum of 53 days, which is a positive start for exploring additional biomaterials to microencapsulate islets, although the response varied from mouse to mouse.

With the achievement of biomaterials, the design of microencapsulated encapsulation has been improved [100,101]. In addition to direct wrapping in a monolayer hydrogel, some researchers have designed an alginate–polylysine (PLL)–alginate (APA) microcapsule, whereby the lysine increases the mechanical properties of the gel, while the outermost layer of alginate attenuates the pro-inflammatory effects of the polycation (Fig. 5B) [102]. Notably, pore size is a critical consideration in microgel design. Although there is no definitive optimal pore size for achieving the desired porosity and molecular weight cutoff, it is generally considered safe to have pores that restrict molecules with a lower molecular weight than the average molecular weight of immunoglobulins, 150 kDa [103]. Based on this, Liu et al. [104] fabricated porous β cell microcapsules utilizing polyethylene oxide (PEO) as a pore-making agent and transplanted them at the omentum of diabetic mice, ameliorating hyperglycemia in the absence of immunosuppressants (Fig. 5C).

In addition to microcapsules encapsulating islet cells, microcarriers are also an important type of microencapsulation. Mantovani et al. [105] seeded dispersed human islet cells on Cytodex1 microcarriers and cultured for a maximum of 8 days. Throughout the studying period, the islet cells proliferated when maintaining their insulin-secreting function. Fang et al. [106] integrated gene therapy with human adipocyte-derived stem cell (ADSC) culture on microcarriers, inducing ADSC into adipocytes. The resultant adipocytes were capable of facilitating insulin expression to alleviate diabetes. Inspired by the natural structure of the stem cell niche, Li et al. [107] prepared a novel porous PEGDA and gelatin methacryloyl (GelMA) microcarrier composite with ECM to mimic the in vivo microenvironment by microfluidic technology (Fig. 5D). The microcarriers loaded with pancreatic β cells were transplanted into diabetic rodents, which demonstrated durable control of blood glucose levels.

Compared to the typical microsphere style of encasing pancreatic islets above, microneedles, as a highly intriguing vehicle in the field of transdermal drug delivery in recent years, have made many attempts at diabetes treatment [108,109]. By loading insulin within the microneedles, Zhao’s group achieved light-responsive release of insulin subcutaneously [110] as well as magnetically responsive release in the intestine [111]. In addition to routine drug delivery by microneedles, in a typical experiment, Ye et al. [112] loaded pancreatic β cells on microneedles and used glucose oxidase, α-amylase, and glucoamylase as glucose-signal amplifiers for cellular delivery to achieve glucose response. Despite the progress made, it is still challenging to maintain long-term islet cell activity after microneedle implantation and to load larger amounts of islet cells, so more exploration of microneedles for diabetic glycemic control remains in the area of drug delivery. In recent years, microneedles loaded with ADSC [98,113] and MSC [114] for diabetic wounds have been attempted in many cases and have made many good advances, which suggests that microneedles encapsulating cells for delivery have a strong potential for the treatment of diabetic complications. All of these provided creative explorations for microencapsulation of pancreatic islet cells.

In general, the advantages of microencapsulation lie in the small size but large surface area to volume ratio, favorable diffusion of oxygen and nutrients, and fast response to changes in serum glucose levels, which allows implantation by minimally invasive surgery and simple handling [115]. Despite the obvious advantages of microencapsulation, its clinical application is subject to several limitations. Due to the large volume required for therapeutic islet cell transplantation, currently available cellular microencapsulation strategies cannot effectively encapsulate sufficient islet cells in a short time. Furthermore, when encapsulating islet cells using micro-devices, the grafts cannot be precisely controlled, making it difficult to retrieve completely.

### Nanoencapsulation

Conventional microencapsulation strategies are usually between 250 and 1500 μm in diameter [116–118]. However, in theory, the ideal oxygen diffusion distance should be less than 100 μm to prevent hypoxia and necrosis of the central pancreatic islet [119]. Therefore, nanoencapsulation may be able to address these limitations by forming a nanoscale barrier layer on the surface of individual islets to encapsulate and protect the cells [120,121].

**Fig. 6.**
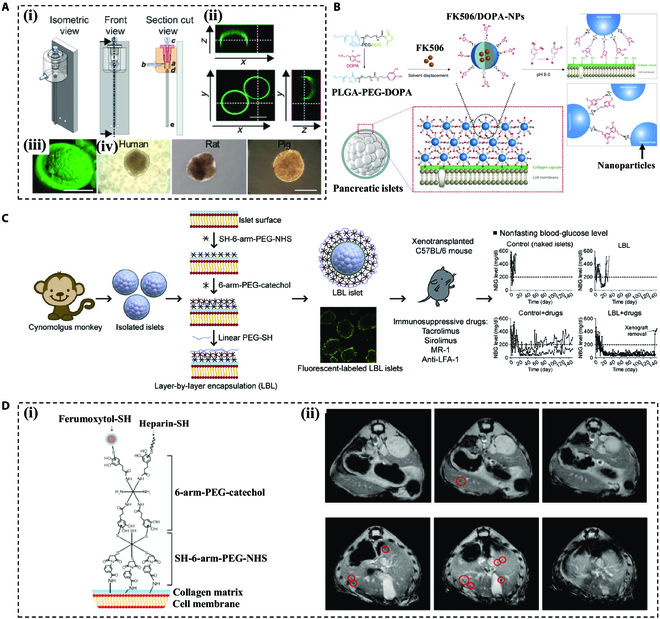
Representative nanoencapsulation devices. (A) Schematic of the device used for islet encapsulation and photographs of encapsulated islets from different species sources. Reproduced with permission [125]. Scale bars, 200 μm (iii) and 100 μm (iv). Copyright 2014, published under the PNAS license. (B) Schematic illustration of surface engineering of pancreatic islet surface by multilayered deposition of FK506/DOPA-NPs. Reproduced with permission [126]. Copyright 2017, Elsevier Ltd. (C) Schematic of LBL nanocoatings encapsulating pancreatic islets and in vivo transplantation. Reproduced with permission [127]. Copyright 2017, Elsevier B.V. (D) (i) Scheme of synthesis and structure of multilayer surface modifications for pancreatic islet MRI using ferumoxytol. (ii) MRI of pancreatic islets encapsulated with ferumoxytol-heparin-PEGylation in a mouse model. Reproduced with permission [128]. Copyright 2019, Elsevier Ltd.

PEG and its derivatives are commonly used biomaterials in nanoencapsulated tissue engineering [122,123]. In the 1990s, Hill et al. [124] harnessed the photopolymerization properties of PEGDA for early exploration of nanoencapsulation. They used a polymer conformal coating to encapsulate pancreatic islets and achieved in vivo graft survival for 1 month in diabetic rats. Tomei et al. [125], by incorporating microfluidics, encapsulated pancreatic islets with a constant PEG coating thickness, providing structural support and protection during in vitro cultivation (Fig. 6A). In addition to the PEGDA, Pham et al. [126] encapsulated pancreatic islets with 3,4-dihydrophenylethylamine (DOPA) and poly(lactic acid-co-glycol)-poly(ethylene glycol) (PLGA-PEG) nanoparticles (DOPA-NPs) carrying immunosuppressant FK506 (FK506/DOPA-NPs) for antigenic camouflage (Fig. 6B). In this system, the encapsulated islets were able to maintain their morphology and viability even after 7 days of coculture with xenogeneic lymphocytes.

In order to expand the range of polymers that can be applied to islet nanoencapsulation, layer-by-layer (LBL) assembly methods that form alternating nanofilms on the islet surface have emerged. Some researchers encapsulated MIN-6 cell spheroids in chitosan/alginate multilayers via the LBL nanocoating technique, and they found that these coatings are effective in shielding MIN-6 cell spheroids from the attack of macromolecules in immune systems [123]. For example, Haque et al. [127] used 3 layers of PEG molecules (SH-6-arm-PEG-NHS, 6-arm-PEG-catechol, and linear PEG-SH) to uniformly encapsulate nonhuman primate islets. In combination with the use of immunosuppressive agents, graft survival of up to 150 days was achieved in vivo, accompanied by low immunogenicity (Fig. 6C). In addition to conventional post-wrap transplantation, Jin et al. [128] expanded LBL to include in vivo magnetic resonance imaging (MRI) tracking and monitoring of grafts. The researchers formed a 4-layer nanoshield around the pancreatic islets using PEG (2 layers), ferumoxytol, and heparin. When transplanted into the portal vein, the islets could be tracked by MRI without compromising islet function (Fig. 6D).

Nanotechnology improves material design, enhances drug delivery, blocks unfavorable protein adsorption, generates or delivers oxygen, and allows precision in specifying molecular weight cutoffs, all of which are key considerations for cellular replacement therapies [129]. However, in terms of clinical practice, the results are not as good as they could be. The only clinical phase 1/2 trial of PEG-encapsulated pancreatic islets for transplantation was previously approved in the United States (Clinicaltrials.gov: NCT00260234). However, no patients were able to achieve insulin independence [130]. Therefore, it remains to be explored how to further optimize nanoencapsulation to make it more compatible with the needs of clinical treatment and bring better safety and therapeutic effects.

Overall, each encapsulation strategy has its own advantages and disadvantages (Table 2). As we mentioned above, of all the encapsulation devices currently available, macroencapsulation is the most commercially available and clinically trial-conducted option, mainly due to its safety in terms of easy retrieval, but huge devices tend to interfere with oxygen and nutrient diffusion to the internal islets [131]. In addition, giant devices can only be transplanted subcutaneously in a majority of cases, which further limit the survival of the encapsulated islets. To address these problems, microencapsulation is preferred. For microencapsulations, the diffusion is greatly improved due to its higher specific surface area so that the bioactivity of the encapsulated cells is maintained and the islets show a faster and more efficient response to blood glucose changes [132]. Based on this, the microencapsulation will be a promising islet transplantation strategy if a rapid and massive microcapsule production system can be developed for clinical applications and the safe retrieval of microcapsules can be ensured as much as possible. As for nanoencapsulation, retrievability is an issue that needs to be addressed urgently [131]. Although it is hard to define which encapsulation strategy has an absolute advantage, researchers can make full use of the benefits of the encapsulation methods to better fit the transplantation demands. Also, more biomaterials are encouraged to turn this advanced islet transplantation into a possible clinical application.

**Table 2.** Summary of different encapsulation strategies.

Strategy	Shapes	Representative materials	Advantages	Disadvantages
Macroencapsulation	Bulk hydrogel	Alginate [70]	Large capacity Mechanical stability Retrievability	Limited diffusion of oxygen and nutrients
		PEGDA [66]		
	Hydrogel sheet	Alginate [71]		
	Hydrogel fiber	Alginate [78,80]		
Microencapsulation	Microsphere	Alginate [95,104]	Large specific surface area Minimally invasive implantation possible	Lack of large and fast preparation systems Difficult to accurately retrieve
		Alginate-polylysine- alginate (APA) [102]		
		Gelma [44]		
	Microcarrier	PEGDA + ECM [107]		
	Microneedle	RGD-alginate + type IV collagen [112]		
Nanoencapsulation	Single-layer	PEG [125]	Short diffusion distances Standardization of coating thickness	Incomplete coating leads to cell exposure Inability to precisely control the position
	Multilayer	DOPA-PLGA-PEG [126]		
		PEG [127,128]		

## Approaches to Improve Transplantation Efficacy

To achieve better islet transplantation therapeutic outcomes, it is not enough to simply discuss different hydrogel encapsulation strategies. Selecting more appropriate graft sites, addressing blood and oxygen supply for long-term islet survival, and mitigating graft rejection are equally critical.

### Optimization of transplantation sites

**Fig. 7.**
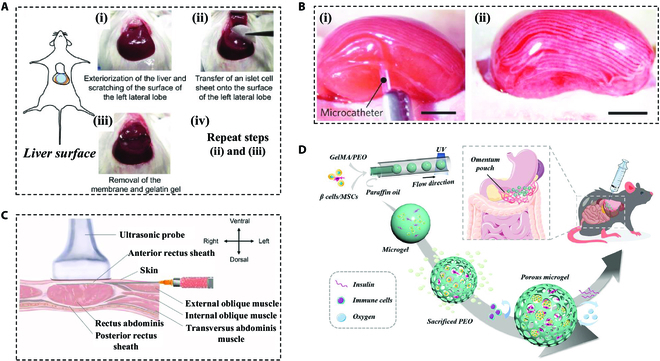
Representative transplantation sites in animal islet transplantation explorations. (A) Schematic of the recovery and transplantation of rat islet cell sheets. Reproduced with permission [138]. Copyright 2018, The Japanese Society for Regenerative Medicine. (B) Images of primary islet cell-laden microfibers implanted into the subrenal capsule cavity of recipient mice. Scale bars, 2 mm. Reproduced with permission [76]. Copyright 2013, Springer Nature Limited. (C) Schematic diagram of ultrasound-guided transplantation of SC-islets in the anterior rectus sheath. Reproduced with permission [141]. Copyright 2023, The author(s), under exclusive license to Springer Nature Limited. (D) Schematic diagram of the porous microgel transplantation in the omentum. Reproduced with permission [44]. Copyright 2023, Wiley-VCH GmbH.

The pancreas, being the physiological site of pancreatic islets, is undoubtedly a crucial consideration for transplantation, but surprisingly few studies have tested islet transplantation in situ. This is mainly due to the difficulty of surgical manipulation in clinical practice, surgical trauma causing leakage of the pancreatic exocrine gland digestive enzymes leading to damage to the graft and surrounding tissues, as well as severe autoimmune reactions within the pancreas in type 1 diabetes, which may inactivate the grafts more rapidly [133]. Since the successful injection of pancreatic islets for transplantation in the rat portal vein in 1973 [134] and in the human portal vein in 1980, the liver has become the most clinically used site for islet transplantation [123,135]. It is manageable for clinical practice to implant islets in the liver, allowing for more insulin circulation. Nevertheless, the direct injection of islets into the vessels can induce an immediate blood-mediated inflammatory response (IBMIR), which causes islet damage and loss of islets [136]. Currently, the ideal islet transplantation site may include the following characteristics: sufficient space to accommodate the graft, easy handling and safe surgical manipulation, adequate blood/oxygen supply, minimal immune/inflammatory response, potential for noninvasive imaging, and biopsy [137]. Fujita et al. [138] found that the surface of the liver is a favorable transplantation site for cell sheets (Fig. 7A). In addition to the liver, the renal capsule has been chosen for transplantation in many animal experiments because of its immuno-isolating properties (Fig. 7B) [76]. However, the site has limited space to accommodate the grafts and be available for invasive procedures, making it unsuitable for clinical use. Similarly, other immune-exempt sites such as the anterior chamber of the eye [139] and the testes [140] are currently only carried out in animal experiments.

Based on the fact that the space between the capsule and the vascularized organ may favor cell survival, growth, and maintenance, Liang et al. [141] hypothesized that the subanterior rectus sheath may be an alternative extraperitoneal site for islet transplantation and they successfully validated this in rhesus monkeys (Fig. 7C). Additionally, recent research has highlighted the omentum as a promising transplantation site [142]. The omentum can accommodate a large number of islet cells, which maintains the satisfying survival of nonpurified islets [143]. Furthermore, it secretes various growth factors such as VEGF and CXC chemokine receptor 4 (CXCR4), which may aid in islet implantation and survival [144]. Several studies have been conducted in rodent and nonhuman primate omentum with naked or hydrogel-encapsulated pancreatic islets transplanted, all of which have realized the glycemic control (Fig. 7D) [44,104,107,145].

Some other sites, like white adipose tissue [146], brown adipose tissue [147], bone marrow [148], spleen [149], muscles [150], and gastric submucosa [151], have also been successfully transplanted with islet cells in animals or some clinical trials (Table 3). Each of the above transplantation sites has its strengths and weaknesses; thus, the most suitable site for diabetic islet transplantation can only be determined by more in-depth studies based on the clinical feasibility, transplantation effect, patient acceptance, and multiple dimensions of each site.

**Table 3.** Clinical trials of islet transplantation at different sites

NCT number	Transplantation sites	Study status	Conditions	Phases
NCT02803905	Omentum	Active not recruiting	Islets of Langerhans transplantation/T1DM	Phase2
NCT01722682	Bone marrow/portal vein	Completed	T1DM	Phase1/phase 2
NCT01345227	Bone marrow	Completed	T1DM/postpancreatectomy hyperglycemia	Na
NCT02213003	Omentum	Active not recruiting	T1DM/hypoglycemia/hypoglycemia unawareness	Phase1/phase 2
NCT01967186	Muscle	Unknown	T1DM/end-stage renal disease	Na
NCT01571817	Gastric submucosa	Completed	T1DM	Phase1
NCT05073302	Subcutaneous	Recruiting	T1DM	Phase1
NCT02367534	Umbilical vein	Completed	T1DM	Phase1/phase 2
NCT02402439	Gastric submucosa	Active not recruiting	T1DM	Phase1

### Improvement of vascularization

The natural pancreatic islet is a highly vascularized organ facilitating oxygen supplement and nutrient exchange and guaranteeing glucose responsiveness [152,153]. Hydrogel encapsulation of cells reduces immunotoxicity to the grafted tissue by preventing contact with immune cells. However, the encapsulation of the cells also prevents the development of intra-islet vascularization [154]. The idealized strategy is to create a vascular system that mimics the natural islet vasculature to improve islet graft performance; however, overcoming the hydrogel barrier may require different solutions [155]. Efforts are underway to develop functionalized encapsulating materials that can attenuate the fibrotic response, induce blood vessel formation, and enhance graft oxygen supply to improve graft survival.

**Fig. 8.**
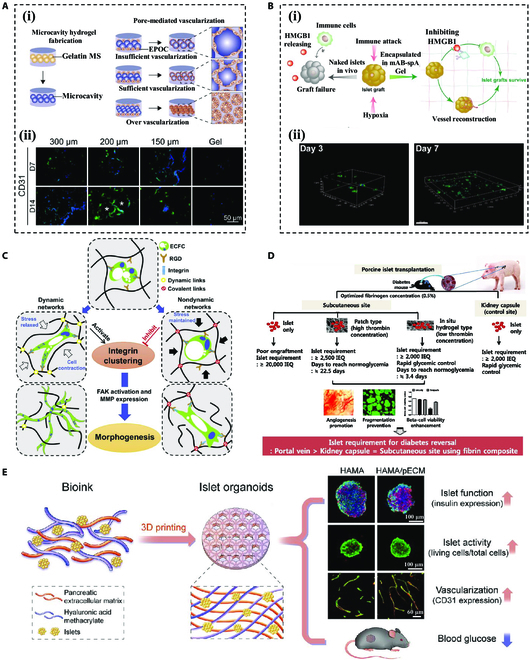
Different strategies to promote graft vascularization. (A) (i) Schematic and (ii) fluorescence graphs of vascularization in hydrogels with different pore sizes. Reproduced with permission [157]. Copyright 2017, Acta Materialia Inc. (B) (i) Schematic illustration of mAB-spA gel promoting in situ immunomodulation and facilitating revascularization in vitro. (ii) Human umbilical vein endothelial cell (HUVEC) proliferation over time in mAB-spA gels. Scale bar, 200 μm. Reproduced with permission [159]. Copyright 2023, Acta Materialia Inc. (C) Mechanism of dynamic hydrogel-mediated assembly of the vascular system and deposition of new basement membranes. Reproduced with permission [161]. Copyright 2020, Elsevier Inc. (D) Schematic diagram of in situ application of hydrogel-type fibrin-islet composites. Reproduced with permission [170]. Copyright 2012, Elsevier B.V. (E) Schematic diagram of schematic of 3D-printed vascularization-assistive pECM/HAMA hydrogel. Reproduced with permission [173]. Copyright 2022, Acta Materialia Inc.

In previous research, it was demonstrated that some inherent properties of hydrogel materials can affect vascularization [156]. Specifically, the pore size of hydrogel not only affects its mechanical properties but also influences the formation of vascular structure (Fig. 8A) [157]. Chiu et al. [158] explored the effect of different pore sizes on vascularization by implanting PEG hydrogels in vivo. Gel vascular implantation with pore sizes of 25 to 50 μm was found to be limited to the outer surface, whereas gels with larger pore sizes (50 to 100 and 100 to 150 μm) allowed the formation of mature vascular tissue throughout the hydrogel. In a recent study, Bai et al. [159] designed a Staphylococcal protein A (spA)-modified hydrogel that captures anti-HMGB1 monoclonal antibody (mAB) (mAB-spA Gel), which inactivates immune cells. It was found difficult to generate blood vessels for too small apertures, whereas the hydrogel with pore sizes over 100 μm promotes angiogenesis via the entrance of vascular endothelial cells (VECs) into the hydrogel (Fig. 8B). In addition to pore sizes, the hydrogel stiffness tends to vary with hydrogel material and composition, which likewise affects the function of leukocytes and angiogenesis [160]. Wei et al. [161] designed a viscoelastic hydrogel system by dynamic covalent cross-linking and found that network dynamics allowed rapid network formation by increasing cell contractility to aggregate integrins (Fig. 8C).

The ECM, as an important microenvironment, is involved in many aspects of vascularization [162]. Encapsulating islets with hydrogel mimicking the natural ECM is an effective strategy to promote vascularization of islet grafts [163,164]. As a major component of the ECM, collagen plays a key role in maintaining islet function [165]. Coculture of islets by pre-vascularized collagen hydrogel markedly improved islet revascularization after transplantation [166,167]. Fibrin, a key protein in angiogenesis, controls endothelial cell behavior and vessel formation through its binding sites with integrins [168]. Consequently, well-organized vascular networks can form around transplanted fibrin hydrogels encapsulating pancreatic islets (Fig. 8D) [169–171], even without pre-vascularization [172]. Another way to promote angiogenesis by ECM is fabricating the hydrogel with decellularized pancreatic tissues, taking full advantage of its natural ECM components. Wang et al. [173] developed a 3-dimensional (3D) printed hydrogel scaffold by utilizing pancreatic decellularized matrix (pECM) and hyaluronic acid methacrylate (HAMA) as a specific bioink, whose in vivo experiments showed excellent vascular network formation (Fig. 8E).

Cytokine delivery is also a powerful group of factors influencing angiogenesis. VEGF is a well-researched pro-angiogenic factor that plays an essential role in promoting vascular endothelial growth in hydrogel [174,175]. The direct wrapping of VEGF into the hydrogel makes the release of VEGF pretty uncontrolled. Attempts to bind VEGF to liposomes [176], to use LBL encapsulation strategies [66], or to prepare a 3D cyclic polycyclic lactone (PCL) scaffold with a heparinized surface to electrostatically bind VEGF [177] have all achieved a controlled release of VEGF and substantial improvements in angiogenesis. Other cytokines such as platelet-derived growth factor (PDGF) and fibroblast growth factor 2 (FGF2) have also demonstrated improvement in angiogenesis and prolonging graft survival when coencapsulated with VEGF.

### Immune regulation

After conventional islet transplantation, immunosuppressants are mandatory medications for recipients to prevent the immune system from attacking and destroying the islets [178]. Although a certain degree of immune isolation was achieved by using hydrogels encapsulating pancreatic islets, further optimization can always be done to achieve stronger immune isolation by modifying the hydrogel material or pancreatic islet cells.

**Fig. 9.**
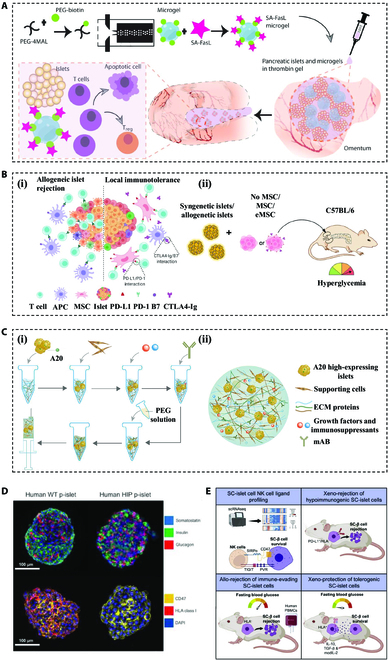
Strategies for improving immunomodulation toward islet transplantation. (A) Schematic representation of synthetic SA-FasL-presenting microgels and their local expression in islet transplantation. Reproduced with permission [145]. Copyright 2022, The Authors. (B) Schematic representation of engineered mesenchymal stromal cells (eMSCs), which induced local immunotolerance. Reproduced with permission [184]. Copyright 2022, The Authors. (C) Schematic of the hydrogel fabrication scheme for a hypothetical islet transplantation model with high A20 expression. “Drug Design, Development 2020:14 4021-4027”: Originally published by and used with permission from Dove Medical Press Ltd. [186]. (D) Immunofluorescence staining of endocrine hormones and immune-related markers of the prepared hypoimmune pseudo-islet. Reproduced with permission [150]. Copyright 2023, The Authors. (E) Schematic diagram of genetically engineered pancreatic islets to promote the generation of a tolerant local microenvironment and treat diabetes. Reproduced with permission [189]. Copyright 2022, Elsevier Inc.

Taking advantage of the sustained-release property of hydrogels, it seems to be a feasible approach to provide adequate local immune protection for grafts by integrating immunomodulatory drugs. The widely used drugs, including glucocorticoids, nonsteroidal anti-inflammatory drugs (NSAIDs), and other immunosuppressive agents, have been employed for hydrogel-encapsulated islet transplantation [179,180]. Dang et al. [181] conducted an in vivo screening of 16 small-molecule anti-inflammatory agents. They suggested that dexamethasone and curcumin were the most effective agents to inhibit the activities of inflammatory proteases and reactive oxygen species (ROS) in the host response to subcutaneously injected biomaterials. Notably, Lei et al. [145] described a strategy for blood glucose control for more than 6 months with short-term rapamycin monotherapy alone. The strategy involves cotransplantation of allogeneic islets and streptavidin (SA)-FasL-presenting microgels into the omentum (Fig. 9A). Combined delivery of interleukin-2 (IL-2) analogs and FasL via hydrogel at the time of islet transplantation by Medina et al. [182] modulated both regulatory and effector T cells as a means of improving the local immunity around the graft. Beyond the above drugs and proteins, the adoption of combined accessory cells [183], such as MSCs (Fig. 9B) [184], and Sertoli cells [185] has also been effective in improving the immune microenvironment.

Another effective immunomodulatory strategy is to edit or modify the transplanted islets themselves. Bai et al. [186] proposed a model of hydrogel encapsulating highly A20-expressing pancreatic islets, which is expected to enable islets to survive longer in vivo (Fig. 9C). Expression of programmed cell death 1 ligand 1 (PD-L1) and indoleamine dioxygenase (IDO) fusion proteins in pancreatic islets by transgenesis has also been shown to inhibit the recruitment of host CD8^+^ T cells and phagocytes as well as the accumulation of FOXP3^+^ recruiting regulatory T cells (T_regs_), weaning recipients from immunosuppressive drug use [187]. In addition, Hu et al. [150] developed a safe and effective hypoimmune graft by editing human primary islets into human leukocyte antigen (HLA) class I- and class II-negative and CD47-overexpressing phenotypes (Fig. 9D). In vivo experiments in both diabetic humanized mouse models [150] and nonhuman primates [188] have demonstrated desirable glycemic control and immune escape effects. For SC-islets, it is similarly possible to express the cytokines IL-10, transforming growth factor-β (TGF-β), and modified IL-2 by genetic engineering, and thus by T_regs_ to modulate the local microenvironment (Fig. 9E) [189].

## Conclusion and Perspectives

Hydrogel-encapsulated islet transplantation for the treatment of diabetes has gradually emerged over the past few decades, regarded as a new treatment modality for diabetic patients away from exogenous insulin injections. Overall, this review divides the hydrogel-encapsulated islet strategy into 3 parts. In terms of cell sources, the main ones include pancreatic β cell lines, primary islets of different species, and various types of SC-islets. Based on the encapsulation size of the hydrogel, it can be categorized into macro-, micro-, and nanoencapsulation. Furthermore, the researchers also selected graft sites and promoted graft vascularization and immunomodulation to prolong graft survival and maintain posttransplant glycemic control.

For the treatment of DM, cell therapy fulfills the therapeutic need to a certain extent, which is expected to restore endogenous insulin secretion in patients and alleviate the discomfort of exogenous insulin injections. However, the shortage of donor sources and the vulnerability of grafts to immune attacks have hindered their widespread use in clinical practices. We believe that the strategy of transplanting pancreatic islets with hydrogel encapsulation is feasible. Through the use of various hydrogels to encapsulate the pancreatic islets, the survival of the cells can be effectively maintained due to the immuno-isolating microenvironment formed by hydrogels. Furthermore, the grafts can survive more durably and play a long-term effect on blood glucose regulation owing to the modification of hydrogels and optimization of transplantation conditions. If a safe and effective hydrogel-encapsulated islet cell transplantation can be successfully realized, it will be a breakthrough in the field of cell therapy and diabetes treatment.

Despite the continuous development of cell therapy and tissue engineering, their research and application in diabetes treatment is still in its infancy. As we have seen, many attempts have been made to encapsulate pancreatic islets in hydrogels in rodents as well as nonhuman primates, and even in some clinical trials for human use. Although with some positive results, encapsulation strategies still need to be refined. First, the hydrogel materials used for encapsulation must be extremely safe and require the ability to be retrieved in time if the graft fails. Second, how to design it to accommodate sufficient therapeutic numbers of islet cells is also something that needs to be considered. As for the transplanted islet cells, if they are primary islets, insufficient sources and allogeneic or homologous rejection are very much to be considered; if they are derived from stem cells, although they are currently considered one of the most promising sources, how to effectively induce them into islet organoids, to improve the induction efficiency and maturity, and to avoid the risk of their unlimited proliferation in vivo need to be explored in depth. Ultimately, improving blood and oxygen supply to the graft, mitigating graft site fibrosis and immune attack caused by the hydrogel itself as an exogenous material, and ensuring the long-term function of the graft in the body are all keys to achieving a successful islet transplantation.

## Data Availability

The data that support the findings of this study are available from the corresponding authors upon reasonable request.
